# Editorial: Deep brain stimulation think tank: updates in neurotechnology and neuromodulation, volume V

**DOI:** 10.3389/fnhum.2025.1609727

**Published:** 2025-04-29

**Authors:** Alfonso Enrique Martinez-Nunez, Sarah-Anna Hescham, Adolfo Ramirez-Zamora, Michael S. Okun, Joshua K. Wong

**Affiliations:** ^1^Norman Fixel Institute for Neurological Diseases, University of Florida, Gainesville, FL, United States; ^2^School for Mental Health and Neuroscience, Maastricht University, Maastricht, Netherlands

**Keywords:** deep brain stimulation, neuromodulation, neurotechnology, Parkinson's disease, epilepsy, obsessive compulsive disorder, stroke

Neuromodulation has been fully integrated into clinical practice since the FDA approved deep brain stimulation (DBS) for the treatment of tremor nearly three decades ago. Using the concepts learned from DBS, such as network-based targeting, biophysical modeling of neuronal stimulation, and electrical stimulation settings, other forms of neuromodulation have been developed and are rapidly expanding and transforming current therapies. Since 2012, the DBS Think Tank has held annual meetings that bring together the leading experts in the field of neuromodulation to discuss future directions and collaborations to exploit novel technologies with the overarching goal of advancing the methods and outcomes of neuromodulation.

This year's XII Think Tank expanded its scope to discuss advances in DBS clinical applications for movement disorders, stroke, traumatic brain injury, sleep, epilepsy, and neuropsychiatric disorders. Discussions included current and emerging devices for DBS, along with novel techniques such as MRI-guided focused ultrasound stimulation and nanomaterial magnetic stimulation. Finally, the ethical implications of chronic device implantation, abandonment, and changes in agency and behavioral patterns were discussed. To reach a global audience of neuromodulation researchers, we publish the proceedings of the meeting each year (Martinez-Nunez, Rozell et al.) and the recordings of the discussions (Deep Brain Stimulation Think Tank, 2024) are made public (with the consent of the lecturers).

In this editorial we provide an overview of the articles included in the fifth volume of the Deep Brain Stimulation Think Tank Research Topic. This Research Topic is published every year with the support of the Frontiers Editorial Office and includes publications on the latest advances in neuromodulation. This volume covers personalized stimulation for epilepsy, troubleshooting DBS for essential tremor, comparing intraoperative stimulation with the monopolar review, and computational models of temporal interference stimulation.

## Personalized stimulation for epilepsy

Suresh et al. described the case of a patient with juvenile myoclonic epilepsy implanted with DBS in the centromedian nucleus of the thalamus (CM) (Suresh et al., p. 20). They studied sleep architecture using asleep electroencephalography (EEG) and compared high stimulation frequency (125 Hz) to low stimulation frequency (10 Hz) during sleep. They found a severely disrupted sleep architecture and a higher seizure burden with high-frequency stimulation. When switching to low-frequency stimulation during sleep, they noted an improvement in sleep architecture organization, better sleep quality, and lower seizure burden.

The stimulation parameters used in DBS for epilepsy were derived from the SANTE trial for stimulation of the anterior nucleus of the thalamus (Fisher et al., 2010). This trial used a standardized stimulation frequency of 140 Hz, therefore the majority of clinical programming is done at these high-frequency settings. The case presented by Suresh et al. exemplifies how potentially engaging different brain networks can lead to different side effects, such as sleep disruption, and this must be addressed on a case-by-case basis.

## Troubleshooting DBS for essential tremor

DBS in the ventral intermedius nucleus of the thalamus (Vim) for essential tremor has increased in efficacy and complexity since its FDA approval. With an increasing number of possibilities for DBS programming, we now have more strategies to maintain good clinical benefit despite gradual disease progression. Some of these strategies include changes in stimulation amplitude and pulse width, changes in omnidirectional contacts, directional stimulation, bipolar stimulation, and interleaving stimulation.

Martinez-Nunez, Sarmento et al. wrote a review for this volume that covers the most common chronic stimulation-induced side effects of Vim-DBS, including dysarthria, dysphagia, ataxia, and gait impairment. The review is summarized with three figures that can be used for reference in the clinic and teaching sessions.

## Comparing intraoperative stimulation with monopolar review in the clinic

It is common to perform a monopolar review in the operating room after a DBS lead is placed to ensure adequate lead position and appropriate therapeutic window to facilitate effective stimulation without the unintended consequence of stimulation-induced side effects (Sammartino et al., 2020). To ensure that the stimulation ranges used in the operating room are comparable to those used in the clinic, the stimulation paradigm must closely resemble the chronic stimulation paradigm used by the implantable pulse generator (IPG) that is connected to the DBS lead.

Mampre et al. compared two different forms of charge balancing: active recharge and passive recharge. Both are methods used to ensure that there is no charge accumulation in the nerve tissue, which could potentially lead to damage. The authors found that the thresholds for stimulation-induced side effects using passive recharge were most similar to those encountered in the clinic when stimulating from the IPG.

Most importantly, they found that both methods of charge balancing resulted in a significant decrease in the stimulation amplitude required to elicit stimulation-induced side effects when compared to monopolar review in the clinic. They found a mean decrease of 0.8 mA for passive recharge, and 1.2 mA for active recharge. This so-called “threshold contraction” is seen in clinical practice and it is often attributed to acute edema around the lead during intraoperative stimulation testing. It usually resolves within a few days following implantation (Borellini et al., 2019). These numbers can provide precise direction for estimating use in clinical programming and shared decision-making, such as whether the expected therapeutic range is good enough, or whether the lead should be repositioned intraoperatively.

## Computational models of temporal interference stimulation

Non-invasive brain stimulation methods are becoming more effective and precise, and several studies have demonstrated significant clinical changes. It has become more important to determine precisely which structures are being stimulated by these techniques. Studies in humans have revealed changes in functional magnetic resonance imaging after transcranial temporal interference stimulation (tTIS), demonstrating modulation of neuronal tissue, although we must appreciate that its spatial resolution and the current intensity needed to effectively modulate brain circuitry remain unclear (Violante et al., 2023).

To explore this phenomenon, Karimi et al. compared the ability of tTIS and transcranial alternating current stimulation (tACS) to modulate a computational neuron model that emulates excitatory and inhibitory neurons. Their modeling of tTIS revealed that superficial brain regions can be stimulated and entrained, and this includes deep brain regions where the current from both stimulation sources overlaps. They also found that tTIS requires a significantly higher current intensity than tACS to entrain the neuronal model. Taken together these findings suggest that when considering a whole-brain model where the target is deep, tTIS has less spatial resolution and less stimulation efficacy than previously concluded, based on single-cell models.

## Conclusion

Advancements in neurotechnology continue to shape and develop clinical management to improve long-term patient care and outcomes. Advances in DBS programming require a precise and personalized approach to lead implantation and programming. Non-invasive stimulation is emerging not only as a potential treatment for neurological diseases but also as a powerful tool for studying brain circuits.

## References

[B1] Borellini L. Ardolino G. Carrabba G. Locatelli M. Rampini P. Sbaraini S.. (2019). Peri-lead edema after deep brain stimulation surgery for Parkinson's disease: a prospective magnetic resonance imaging study. Eur. J. Neurol. 26, 533–539. 10.1111/ene.13852 30358915

[B2] Deep Brain Stimulation Think Tank (2024). University of Florida. Available online at: https://fixel.ufhealth.org/think-tanks/deep-brain-stimulation-think-tank/ (accessed November 26, 2024).

[B3] Fisher R. Salanova V. Witt T. Worth R. Henry T. Gross R.. (2010). Electrical stimulation of the anterior nucleus of thalamus for treatment of refractory epilepsy. Epilepsia 51, 899–908. 10.1111/j.1528-1167.2010.02536.x 20331461

[B4] Sammartino F. Rege R. Krishna V. (2020). Reliability of intraoperative testing during deep brain stimulation surgery. Neuromodulation 23, 525–529. 10.1111/ner.13081 31823438

[B5] Violante I. R. Alania K. Cassarà A. M. Neufeld E. Acerbo E. Carron R.. (2023). Non-invasive temporal interference electrical stimulation of the human hippocampus. Nat Neurosci. 26, 1994–2004. 10.1038/s41593-023-01456-8 37857775 PMC10620081

